# Design of RNAs: comparing programs for inverse RNA folding

**DOI:** 10.1093/bib/bbw120

**Published:** 2017-01-03

**Authors:** Alexander Churkin, Matan Drory Retwitzer, Vladimir Reinharz, Yann Ponty, Jérôme Waldispühl, Danny Barash

**Affiliations:** 1 Shamoon College of Engineering and Physics Department at Ben-Gurion University, Beer-Sheva, Israel; 2 Department of Computer Science, Ben-Gurion University, Beer-Sheva, Israel; 3 School of Computer Science, McGill University, Montréal QC, Canada; 4 Laboratoire d’informatique, École Polytechnique, Palaiseau, France

**Keywords:** RNA design, inverse RNA folding

## Abstract

Computational programs for predicting RNA sequences with desired folding properties have been extensively developed and expanded in the past several years. Given a secondary structure, these programs aim to predict sequences that fold into a target minimum free energy secondary structure, while considering various constraints. This procedure is called inverse RNA folding. Inverse RNA folding has been traditionally used to design optimized RNAs with favorable properties, an application that is expected to grow considerably in the future in light of advances in the expanding new fields of synthetic biology and RNA nanostructures. Moreover, it was recently demonstrated that inverse RNA folding can successfully be used as a valuable preprocessing step in computational detection of novel noncoding RNAs. This review describes the most popular freeware programs that have been developed for such purposes, starting from RNAinverse that was devised when formulating the inverse RNA folding problem. The most recently published ones that consider RNA secondary structure as input are antaRNA, RNAiFold and incaRNAfbinv, each having different features that could be beneficial to specific biological problems in practice. The various programs also use distinct approaches, ranging from ant colony optimization to constraint programming, in addition to adaptive walk, simulated annealing and Boltzmann sampling. This review compares between the various programs and provides a simple description of the various possibilities that would benefit practitioners in selecting the most suitable program. It is geared for specific tasks requiring RNA design based on input secondary structure, with an outlook toward the future of RNA design programs.

## Introduction

The inverse RNA folding problem for designing sequences that fold into a given RNA secondary structure was introduced in the early 1990’s in Vienna [1]. Mathematically, much like the typical situation with inverse problems, it is not a well-posed problem by the standard definition of Hadamard, which makes it even more challenging to solve. As the well-known mathematician Andrey Tikhonov once noted, the class of ill-posed problems includes many classical mathematical problems and, most significantly, that such problems have important applications. Indeed, new emerging subfields that are of significant importance to a variety of functional RNAs [2–6], such as RNA synthetic biology [7, 8] and RNA nanostructure [9, 10], are fast developing and are using in their arsenal the methods for solving inverse RNA folding [11–18].

A brute force approach that searches all the possible sequences is not a viable option because the number of sequences grows exponentially as 4^*n*^, where *n* is the length of the sequence, while the number of valid designs can be arbitrarily small. This upper bound can be refined by noting that paired positions have to form valid base pairs under the standard A-U, C-G, G-U base pairing scheme. This implies that 6^*p*^^/2^4^*u*^ sequences are compatible with a secondary structure having, respectively, *u* unpaired and *p* paired nucleotides. As a practical consequence, a typical 74-nucleotides-long tRNA, including *p* = 40 paired and *u* = 34 unpaired ones, would require investigating ∼10^36^ compatible sequences.

RNA inverse folding also has deep connections with theoretical evolutionary studies, where the sequence/structure relationship in RNA is a popular model for studying genotype/phenotype maps [19, 20]. For instance, the identification of undesignable motifs [21] in empirical design studies immediately implies that only a negligible, exponentially decreasing on the length, proportion of secondary structures can be designed. Conversely, neutral evolution provides theoretical foundations for the practice of RNA design, and studies of the neutral network confirm a highly variable numbers of admissible designs within structures of the same length [22]. It is interesting to note that the distribution of the neutral network [19] could help us understand how to further develop efficient local search strategies to reach the target structure.

**Figure 1:**
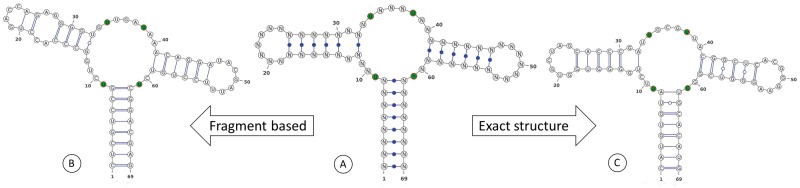
The standard inverse RNA folding problem and the generalized inverse RNA folding problem that is shape aware and fragment-based (i.e. fragment selection enabled) are illustrated on the purine riboswitch aptamer in the middle (**A**). The predicted structure of an output designed sequence is shown on the right (**C**) for the standard inverse folding problem and on the left (**B**) for the generalized inverse RNA folding problem.

The approach to solve the inverse RNA folding problem by stochastic optimization relies on the solution of the direct problem using software available in RNA folding prediction Web servers, e.g. the RNAfold server [23, 24] or mfold/UNAFold [25, 26] as well as RNAstructure [27], by performing energy minimization with thermodynamic parameters [28, 29]. It should be noted that in principle, although far less popular in practice in the context of inverse RNA folding, other programs based on probabilistic models and posterior decoding that have been benchmarked in [30], e.g. Pfold [31] and CentroidFold [32], can also be used to solve the direct problem. Initially, a seed sequence is chosen, after which a local search strategy is used to mutate the seed and apply repeatedly the direct problem of RNA folding prediction by energy minimization. Then, in the vicinity of the seed sequence, a designed sequence is found with desired folding properties according to the objective function in the optimization problem formulation. Chronologically, algorithmic improvements that relate to this approach, which was pioneered in Vienna’s RNAinverse [1], have been worked out in INFO-RNA [33], RNA-SSD [34] and NUPACK:Design [35, 36]. Alternative methods to the adaptive random walk [1] and the stochastic local search [33, 34] include genetic algorithms (belonging to the class of evolutionary algorithms) [37–39], an improved evolutionary algorithm [40, 41], constraint programming [42, 43] and ant colony optimization [44, 45]. On the methodological side, two separate ideas that were gradually developed and investigated were to sample the sequence space more efficiently and to include user-selected fragments in the design. The first idea started in [33] and then by presenting a global sampling approach named RNA-ensign [46], followed by a weighted sampling algorithm called incaRNAtion [47] that is a glocal methodology combining the global sampling approach with local search strategies. The second idea started by generalizing the inverse RNA folding problem to include RNA designed sequences that are predicted to fold into a prescribed shape [37, 48], a utility named RNAexinv that considers the coarse-grain tree graph [49] for shape representation (or potentially abstract shapes [50]). It culminated with a method called RNAfbinv [51] that allows a user-selected prescribed fragment to be preserved exactly by secondary structure (in addition to its shape), whereas the rest of the structure is designed by the generalized shape-based approach. These two ideas were recently merged into an RNA design Web server called incaRNAfbinv [52] that provides more flexibility in the design as compared with the aforementioned methods. An example of the benefit of incaRNAfbinv is illustrated in Figure 1 where it is shown that the designed sequence on the left (Figure 1B) is a feasible purine riboswitch candidate as much as the designed sequence on the right (Figure 1C), both containing the essential nucleotides for either guanine or adenine binding, but the Figure 1B solution cannot be reached to-date by other programs besides incaRNAfbinv because its secondary structure is different than the input structure depicted in Figure 1A, although its tree graph shape is the same.

As was mentioned above and exemplified in the shape aware capability, different computational frameworks for the inverse RNA folding have been implemented in the various programs. In addition to the strict definition that restricts solutions to those sequences whose minimum free energy structure is exactly the target structure, more relaxed frameworks like ensemble defect optimization in NUPACK have been introduced and developed [35], in addition to minimizing the classical cost function given by the ‘structure distance’ between the structure of the test sequence and the target structure [1]. For a candidate sequence and a given target secondary structure, the ensemble defect is the average number of incorrectly paired nucleotides at equilibrium evaluated over the ensemble of unpseudoknotted secondary structures [36]. It could also be possible, although not currently done by any available software, to add prescribed kinetic properties (fast folding and absence of kinetic traps) to the objectives of design, as explored in earlier studies [35]. As will be noticed in the quantitative comparison performed in the continuation, these different computational frameworks will also make it impossible to draw a conclusion as to which program is better for use based on a benchmark. Instead, when facing an application that requires inverse RNA folding, the goal is to try and identify which is the most suitable program for each case.

Some inverse RNA folding programs are geared toward more specific biological problems, compared with the ones mentioned up until now that are general in their application scope. For example, nanostructure design including pseudoknots was performed with Nanofolder for the case of multistranded RNA secondary structure [12]. In another example, for designing the most stable and unstable messenger RNA sequences, which code for a target protein, an algorithm was developed in [53]. On a similar topic, in [54], an algorithm for designing a protein-coding sequence with the most stable secondary structure called CDSfold is provided. For designing multiple target artificial miRNAs, a Tabu search was used in conjunction with biochemical considerations in [55]. For designing RNA sequences that fold into multiple target structures, which makes it possible to efficiently design multi-stable RNA sequences, a program called RNAdesign was introduced in [56]. Another specialized application, given here for completeness, is to allow game players to propose a set of rules for RNA design, as part of the Eterna project. Based on experimental results, Eterna players came up with a set of design rules, and EternaBot was developed to design a sequence based on those rules [57]. Finally, for the problem of fixed backbone three-dimensional design of RNA, the Web server RNA-redesign was put forth [58]. To the best of our knowledge, this is the first implementation of an RNA design program that considers tertiary structure, although it is a local design. In this respect, in secondary structure, sequences that are generated by point mutations performed on an input sequence to optimize a certain objective function may also be considered a design procedure in the weaker (local) sense. Example of such programs are RNAmutants [59] that uses an efficient sampling procedure based on the Boltzmann-weighted ensembles of mutants, and RNAmute [60] that uses suboptimal solutions of the RNA free energy minimization prediction [61, 62] for simulating only selective mutations from all possible ones. These procedures could potentially also be integrated into RNA design tools in the future, as was already done by incorporating RNAmutants ingredients into incaRNAtion [47].

Inverse RNA folding programs are not merely used for the design of artificial sequences to mimic natural ones. Recently, they have been used for the detection of novel naturally occurring RNAs as a preprocessing step before sequence-based searches [63, 64]. In both of these separate works, they have shown to find attractive candidates for naturally occurring RNAs that are not available in Rfam [65] and are missed by standard methods for RNA detection. This approach can well contribute to ongoing efforts aiming for *de novo* discovery of structured noncoding RNAs in genomic sequences [66].

## Details of use

Installing the different programs or accessing the Web servers is not a difficult task. They all contain a ReadMe file or a manual that is easy to follow with no prior knowledge assumed. However, not all programs provide both Web server and source code. Table 1 lists the various RNA design programs according to four categories: general purpose programs, shape aware programs, adaptive sampling programs and specialized programs. It then indicates the availability of Web server or source code for each program, including extended features and general remarks that relate to their capability, use or strategy without providing more details on their specific methodology.

**Table 1:** A Tabular overview with some basic information about the various RNA design programs

Programs	Webserver	Source code	Extension to pseudoknots	Multitarget capability	Remarks
General					
AntaRNA [44, 45]	✓	✓	✓		
RNAiFold [42, 43]	✓	✓		✓	Experience in biology ‘wet laboratory’
RNAinverse [1]	✓	✓			First program developed; experience in biology ‘wet laboratory’
NUPACK [35, 36]	✓	✓			Optional multistranded target structures; experience in biology ‘wet laboratory’
INFO-RNA [33]	✓	✓			
RNA-SSD [34]	✓				
Frnakenstein [39]		✓		✓	
ERD [40, 41]	✓	✓	✓		
MODENA [38]		✓	✓	✓	
Shape aware					
IncaRNAfbinv [52]	✓	✓			Fragment selection enabled; experience in RNA detection
RNAfbinv [51]		✓			Fragment selection enabled
RNAexinv [48]		✓			No user-selected fragment
Adaptive sampling					
IncaRNAfbinv [52]	✓	✓			Global–local approach; experience in RNA detection
IncaRNAtion [47]		✓			Global–local approach
RNA-ensign [46]		✓			Global approach
Specialized					
Nanofolder [12]	✓		✓		Nanostructures; multistranded RNA; experience in biology ‘wet laboratory’
CDSfold [54]	✓	✓			Design of protein-coding sequence
RNAdesign [56]		✓		✓	
EternaBot [57]	✓				Design rules set by Eterna players
RNA-redesign [58]	✓				Three-dimensional: fixed backbone

From all programs listed in Table 1, we picked five programs for further description on their details of use based on the following selection criteria: the programs have both a Web server and a source code available, and they were already used in the literature in a biological meaningful way by either a ‘wet laboratory’ experiment or the identification of a putative new noncoding RNAs. These criteria are of interest to practitioners who are considering the use of RNA design programs. The selection yielded the programs RNAinverse, RNAiFold, NUPACK and incaRNAfbinv. In addition, the antaRNA program was added because it was published recently without a chance yet for practical use, but it is considered promising as can also be observed by the program comparisons provided in next section and its overall strategy that allows much flexibility. Finally, although Nanofolder could not be added because of a lack of source code and our recommendation for the interested user would be to contact its authors [12], it is worthwhile noting the significant practical experience that has been accumulated by Nanofolder as a specialized program for RNA nanostructure design. There are sequence design rules implemented in Nanofolder that have been formulated based on the concept of same-length sequence fragments called ‘critons’ [12], which have been successfully applied beforehand to the design of DNA nanostructures. These special rules with used penalty-scoring terms have been formulated to avoid unstable RNA designs and optimize the designed sequences.

User experience with the five selected programs was performed on two example input secondary structures in dot-bracket notation (the first is a toy problem, an artificial structure; the second is the structure of the guanine-binding riboswitch aptamer, a natural structure):

1. ((((…(((….)))…((((….))))…))))
2. ((((((((…(.(((((…….))))).)…….((((((…….)))))).))))))))

### RNAinverse in detail

RNAinverse [1] was the first program developed for RNA design. It is available as a Web server at http://rna.tbi.univie.ac.at/cgi-bin/RNAinverse.cgi and as a stand-alone version in the Vienna RNA package [24]. The algorithm uses an adaptive random walk to minimize base pair distance. The distance is calculated by comparing the minimal energy folding of a mutated sequence (its predicted structure) with the target structure. To avoid folding the entire sequence, small substructures are optimized and then elongated. The algorithm also supports designing sequences, which are more probable based on the partition function. Those sequences may be more stable but mostly differ from the target structure.

The server receives a secondary structure in dot-bracket notation. An optional start sequence can be inserted; any lower-case letter in the sequence will be conserved in the final result. The server also supports multiple energy models, folding temperature and number of sequences to generate. Once the form is submitted, the result page will appear with a list of designed sequences and the calculated minimum energy for them. It may also show designed sequences that did not match the exact structure and their base pair distance away. Another set of results that will appear below that are designed sequences using the partition function if selected. The Web server also shows links to RNAfold [24] for extensive information on a specific result. The command line used to run the design in the stand-alone version is also written.

The stand-alone version of RNAinverse is part of the Vienna RNA package. The package is a C code library that includes several stand-alone programs. This means RNAinverse can be accessed both from command line and through the package API. The simple API allows the user to design and test the sequences for individual purposes directly through C. The stand-alone version is simple, and examples can be generated by using the Web server as discussed above.

### RNAiFold in detail

RNAiFold [42, 43] is a well-established program available as a Web server at http://bioinformatics.bc.edu/clotelab/RNAiFold/. It uses constraint programming and is available in two modes. RNA-CP design takes as simplest input a secondary structure and returns up to 50 sequences or the maximum found in 2 h, optimized for one of three different criteria: yielding the target as the minimum free energy (MFE) structure, the free energy or the ensemble defect. RNAiFold ensures the optimality of the solutions. Additional constraints can be provided such as a target amino acid sequence, and limits on the amount of GC, AU and GU base pairs as a list of admissible and forbidden base pair. The nucleotide distribution and energy model are customizable. A special mode, RNA Synthetic Design, leverages RFAM [65] to add constraints from sequence conservation. A drop-down menu allows the selection of any family and automatic extraction of the constraints. The consensus structure can be automatically selected as target. The constraints from RNA-CP design are also available.

The output presents for each resulting sequence a number of different statistics such as its GC content, energy and entropy and its amino acid sequence. It also provides a link to BLAST [67], the resulting sequence. Structure (2) was tested on the Web server. It took a short time before the query was allowed to run and returned 23 sequences after 2 h. A version can be downloaded in source code or binaries for Linux or Mac OS X; the source requires the Vienna Package as the open source Google optimization library OR-Tools. The binary was tested on Ubuntu 12.04, while the Mac binaries still need to be updated for OS 10.11.5. It provides a simple input by command line argument or through a file in a custom format to fine-tune a few more arguments of the algorithm itself, and provides a similar output.

### AntaRNA in detail

AntaRNA [44, 45] is a recent program available since 2015, as a Web server at http://rna.informatik.uni-freiburg.de/AntaRNA/. It uses ant colony optimization to allow the design of structures, allowing pseudoknotted structures using hardcoded energy parameters. A sequence constraint can be provided in the IUPAC format. A visualization of the secondary structure with the sequence constraint is dynamically shown. Additionally, a target GC constraint can be set. The parameters of the ant colony algorithm can be modified through advanced options.

Each output sequence, up to a 100, is shown with its MFE structure and its distance from the target GC, target structure and target sequence. A click on a sequence will show a visualization of its secondary structure with the sequence embedded. Testing for Structure (2) while requesting 100 sequences took a few minutes on the Web server. All the results contained all the requested base pairs, a few additional base pairs were sometimes present. A convenient link to download all the sequences in FASTA format with or without their MFE is provided.

The python script requires the Vienna RNA Package and provides the same options as the Web server. For pseudoknots prediction, a finer control is provided requiring the user to have installed one of the programs RNAshapes studio [68] or HotKnots [69] or IPKnot [70]. All the options from the Web server are present and must be given through command line arguments. The script removes the limit of sequences sampled.

### NUPACK in detail

NUPACK [35, 36] is a recent program developed in 2011. It is available as a Web server at http://www.nupack.org/design/new. Its objective is to minimize the ensemble defect for a pseudoknot-free structure. Its interface allows the user to specify a target secondary structure as a preference for DNA or RNA. An interesting feature of NUPACK is the ability to define unwanted motif by providing a list of forbidden sequence motifs, in IUPAC format. The Web server allows to choose between two energy models, Turner95 and Mathews99, dangles and setting the temperature. A maximum of 10 sequences can be designed concurrently.

The output presents the designed sequences. Tested on Structure (2), in a few minutes, 10 sequences were generated. An analysis of the sequences can be launched immediately to compute the MFE and base pair probabilities. A range of temperature can additionally be provided for this step. A link is provided to download the MFE secondary structure representation and the base pair probabilities.

NUPACK is also available as command line software and was tested on a MAC OS X 10.11.5. Some options are not available on the Web server, such as setting a seed sequence or specifying the concentration of salt and magnesium. A few parameters of the algorithm can also be modified from the command line as its random seed, which is necessary to generate different sequences.

### IncaRNAfbinv in detail

IncaRNAfbinv [52] is a recent program for fragment-based design. It is available as a Web server at https://www.cs.bgu.ac.il/incaRNAfbinv/. The Web server combines two base applications: IncaRNAtion [47] and RNAfbinv [51]. Both applications are available as a stand-alone client. RNAfbinv uses simulated annealing with a four-nucleotide look ahead local search function. The function includes biologically meaningful constraints such as sequence constraints, fragment-based design and a variety of optional features. The resulting sequences fit a coarse-grained tree graph shape of the original target structure, thus allowing for flexibility. IncaRNAtion augments the local search method. It uses a global sampling approach and weighted sampling techniques. The sequences generated by IncaRNAtion are used as seed sequences for the local search. The incorporation of those seeds forces highly distributed results and better control of GC content.

The Web server takes as input the target secondary structure in dot-bracket representation. It then converts it to coarse-grained tree graph shape for future comparison. After inserting a structure, an image will appear, as well as a list of structural motifs from which the user can select a desired motif. Additional optional parameters are sequence constrains, target fold energy, mutational robustness and GC content. Submission of the query leads to a Web page showing design progress. Once all the results are ready, a list will appear with the designed sequences as well as their predicted structure, folding energy, mutational robustness, GC content and distance to the original structure. The distances are calculated for both base pairs and structural motifs. Finally, a link is available for an image of the designed structure.

The stand-alone versions of both tools can be run locally; links can be found in the Web server. IncaRNAtion is a simple to run Python script, thus requiring Python installed and recommends adding the MPMATH library for long sequences. It receives as input a file containing the target structure and an optional multiple sequence alignment (MSA). The user is also required to enter a value between 0 and 1, where 1 means only to regard the structure and 0 the MSA. Additional variables are available for GC content control and specific sequence constraints. A single run will generate a large amount of sequences; a minimal value for the number of outputs can also be set as input. The output is a list of sequences separated by lines.

RNAfbinv is a C application, but it is also available wrapped in a Java interface. Once the Java application is running, the user must first insert the secondary structure. The user then has an option to select a specific motif to preserve as is. After inserting the structure, the user arrives to a new screen where additional control variables are available such as target folding energy and mutational robustness. The results appear in a new screen as a list including the base pair distance from the input structure.

### Using the programs

The main task for using these programs is to insert an RNA secondary structure into one of them and generate sequences accepting this target structure as the MFE structure, with possible generalizations that are closely related to this framework. All programs offer the possibility of additional parameters to be chosen by the user, with default values displayed at the beginning. Some programs offer more flexibility in their constraints than others, which is indicated in Table 1 for some selected features that are shared by several programs and are general in scope. As final output, all programs offer description of parts of the analysis of designed sequences as well. RNAiFold displays the results in one Web page per solution, seemingly in the order generated and selectable through a drop-down menu. It presents the MFE structure of each sequence, as an ensemble of statistics, and provides an option to BLAST them. AntaRNA directly displays each generated sequence with its MFE structure. A click on each of the solutions creates a figure of the secondary structure with the sequence. Any constraint violation is represented in red in the figure. The list of sequences can be downloaded in FASTA. NUPACK presents each sequence by increasing ensemble defect. Each sequence has a link to the analysis tool, which computes the MFE structure and base pair probabilities. In addition, the textual output provided in all these programs is substantially contributing to the analysis, as an essential step before the graphical output. In general, most of the programs are user-friendly for the novice. Especially, the programs that have a Web server capability (Table 1) can also be worked out by a nonspecialist user along with the corresponding manuals and instructions that are available in the Web sites.

## Discussion

The programs listed in Table 1 have been developed in the past several years, following the first program named RNAinverse that was introduced >20 years ago, and offer some interesting prospects for RNA sequence design. They all in one way or the other rely at present on thermodynamic parameters corresponding to the nearest-neighbor model and therefore structures that are known to be well predicted by energy minimization, for example the secondary structure of the guanine-binding riboswitch aptamer that is illustrated in the second test case example of previous section and in Figure 1, are the best to work with as inputs to these programs to achieve reliable results. Though exceptional cases exist, in general, the upper range estimate for the sequence length that these programs are useful for is around 150 nucleotides [71]. It is expected that in future, having more experimental structures elucidated, the number of RNA sequences with a well-predicted secondary structure by energy minimization techniques will grow significantly, and more biological systems involving RNAs will be designed by the aid of these programs.

**Figure 2:**
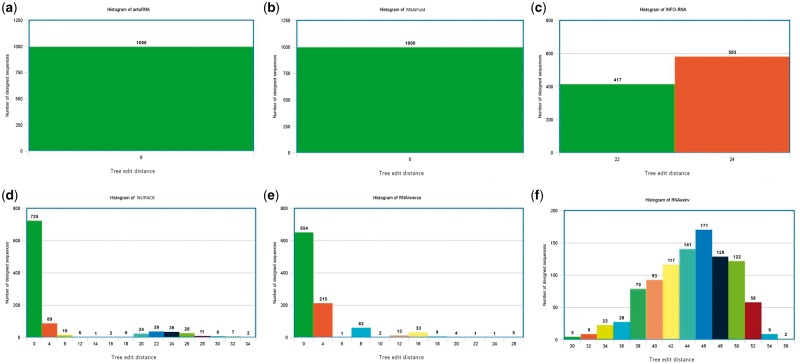
Histogram comparison between the six selected programs is available in Table 2 for the example test case that is designated as (1) in the Details of Use Section and for which the runtimes are reported in the first column of Table 2.

**Figure 3:**
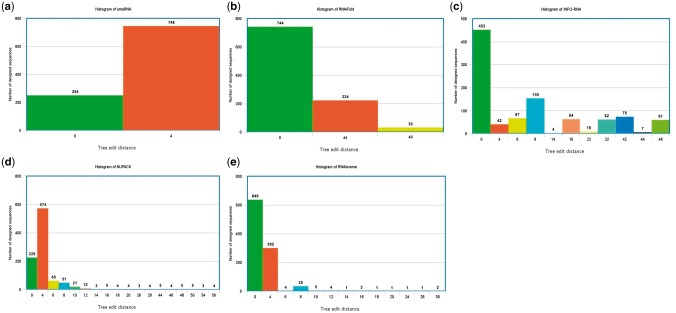
Histogram comparison between the five selected programs is available in Table 2 for the example test case that is designated as (2) in the Details of Use Section and for which the runtimes are reported in the second column of Table 2.

Runtime can be a critical issue concerning the usage of these tools. A runtime comparison of six programs is provided in Table 2 for the two test case examples that were provided in the previous section in dot-bracket notation (the first is a toy problem and the second is the structure of the guanine-binding riboswitch aptamer). The times reported are in minutes. Standard parameter values were used in the comparison. Because runtimes are measured in downloadable source code and cannot be measured in programs that require user interactive intervention such as with incaRNAfbinv and RNAfbinv, we replaced incaRNAfbinv that was discussed in the previous section by the simpler and less developed program RNAexinv. The justification is that RNAexinv is shape aware (preserving the same coarse-grain tree graph shape in the output as in the input) without the user’s interactive selection of a fragment for preserving its secondary structure exactly like in incaRNAfbinv; therefore, RNAexinv contains the shape aware feature itself for inclusion in the comparison. RNAexinv is still much slower than the rest of the programs because it solves a more general inverse RNA folding problem that is shape aware. By our past experience, the programs RNAfbinv and incaRNAfbinv are about 10% more computationally expensive than RNAexinv. Additionally, we inserted the program INFO-RNA because it is known to be the most computationally efficient among all programs, as is also observed in Table 2. Correspondingly, for the two test cases measured in Table 2 by 1000 runs, a histogram is plotted in Figure 2 for the first test case, and in Figure 3, for the second test case to examine how far away the predicted structures of the designed sequences are from the input structure that is initially given. The distance between the two secondary structures was measured by the RNAdistance routine available in the Vienna RNA package that calculates by default the tree edit distance.

**Table 2:** Runtimes for six selected programs (1000 runs for each of the two test cases)

Program	**Time (in minutes)**— Figure 2	**Time (in minutes)**— Figure 3
antaRNA	6.5	7.8
RNAiFold	41.4	6.4
INFO-RNA	0.5	0.8
NUPACK	37.5	217
RNAinverse	3.15	3.7
RNAexinv	231	N/A

While it is impossible to draw conclusions from Table 2 and the associated Figures 2 and 3 as to which program is better for use, because a program such as RNAexinv belonging to the shape aware category (Table 1) is expected to be much slower along with histograms that are more widespread compared with the rest of the programs in a notably beneficial way for its purpose, some trends can be observed. For example, INFO-RNA is the quickest as expected, but its histograms are more widespread compared with most of the other programs and this correlates with the known result that sequences designed with INFO-RNA tend to be more biased to high GC contents [47]. In contrast, the newly introduced antaRNA program from the same laboratory is both relatively fast and achieving histograms with results that are close to the input structure. RNAiFold is also showing some fairly balanced outcomes between efficiency and proximity of the results to the input structure. Finally, RNAinverse shows impressively that although it was written >20 years ago and it features less constraints compared with the newer programs, it is still both fast and faithful to the input structure.

The above comparison is by no means exhaustive and can be supplemented by the additional references [43–45, 52]. These references could benefit a reader interested in the topic of runtime comparisons, design capabilities and properties of the output sequences produced by each method. The RNAiFold Web server article [43] contains a section on comparison with other software. Because this article does not include the most recent methods antaRNA and incaRNAfbinv, the interested reader can find more information about the performance of these algorithms in [44, 45] and [52], respectively. It should also be noted that the use of tree edit distance to the target structure in Figure 3 as a performance measure may not consider that some of the methods included do not necessarily use the same energy model and dangle treatment as used herein. The one used herein for computing the tree edit distance is the Turner 2004 model [29] included in the Vienna RNA package 2 [24]. While NUPACK results could be moderately accurate in any case, as ensemble defect optimization mitigates the slight differences between energy models, the results of RNAinverse, RNAiFold, INFO-RNA and the rest of the programs could be affected by the energy model of choice.

### New prospects: designed RNAs for structure-based search

As was mentioned in the Introduction, a major new application of inverse RNA folding programs is the discovery of novel, structured and functional RNAs in transcriptomic data. We briefly describe the concept and refer the interested reader to [63, 64] for more information.

Sequence-based search tools like BLAST [67] have been used extensively for the detection of novel RNAs of interest, such as riboswitches, in newly sequenced data. They are easily available, highly efficient and can partially address this task. However, when the search is restricted to only sequence-based considerations, it is rather limited. The idea to augment BLAST search with inverse RNA folding for including structure-based considerations has been developed independently for identifying IRES-like structural subdomains [63] and riboswitch aptamer domains [64], where in the first reference, the findings were also verified experimentally, and in the second reference, the experimental verifications are ongoing. In both of these works, this strategy has been shown to yield attractive candidates that are beyond the reach of well-established methods like Infernal [72]. Consequently, an idea was even suggested by the authors of Infernal to augment their own tool by the inverse RNA folding preprocessing step. Various combinations should be tried, and in any case, it is expected that in the future, inverse RNA folding would become useful not only for the design of synthetic RNAs but also for the search of naturally occurring RNAs by the use of designed RNAs as a preprocessing step.

### Concluding remarks

The various programs, especially the ones who are gaining experiences in biological meaningful problems and are being improved as a consequence by updated versions, should best be examined along with the constraints they allow and their orientation purposes. There are already several programs that were described in detail and offer both a Web server implementation and source-code availability, along with a proven experience in biological meaningful problems. Other programs should strive to achieve these goals. Practitioners should then select which program is more suitable for their needs according to the specific constraints and capabilities that are advertised in each one of the programs.

**Key Points**

- RNA design programs should be made user-friendly and accessible to biologists as much as possible, both in terms of ease of use and simplification of the input and output such that it becomes understandable to the nonspecialist.
- In most cases, a balanced trade-off between efficiency and performance in terms of the quality of the designed sequences would be the best option for the design.
- From the algorithmic standpoint, the weighted sampling approach to sample the sequence space efficiently and the fragment-based design approach are desired directions that can be further developed to yield more flexibility in the design procedure.
- Programs for RNA design should aim to accumulate practical experience in biological meaningful problems, be it experimental design or computational searches for novel noncoding RNAs.

## Supplementary Data

Supplementary data are available online at http://bib.oxfordjournals.org/.

## Supplementary Material

Supplementary Data

## Notes

**Alexander Churkin** is a computational scientist and a lecturer at the Shamoon College of Engineering. His research interests include computational biology, RNA structure predictions and scientific computing.

## Notes

**Matan Drory Retwitzer** is a computational scientist who is pursuing his PhD studies in the Computer Science Department at Ben-Gurion University. His research interests include computational biology, RNA structure predictions and RNA structure probing by experimental methods.

## Notes

**Vladimir Reinharz** is a computational scientist and a PhD graduate of the School of Computer Science at McGill University. He is the recipient of an Azrieli and FQRNT postdoctoral fellowships to be pursued at Ben-Gurion University. His research interests include computational biology, RNA structure predictions and scientific computing.

## Notes

**Yann Ponty** is a computational scientist and a faculty member of the bioinformatics group in the Computer Science Department (LIX) of École Polytechnique. His research interests include computational biology, RNA folding and visualization combinatorics and discrete algorithms.

## Notes

**Jérôme Waldispühl** is a computational scientist and a faculty member in the School of Computer Science at McGill University. His research interests include computational biology, RNA and protein structure predictions, as well as crowd computing systems.

## Notes

**Danny Barash** is a computational scientist and a faculty member in the Computer Science Department at Ben-Gurion University. His research interests include computational biology, RNA structure predictions and scientific computing.
