# Supplementary material for: Design of RNAs: comparing programs for inverse RNA folding
Source: Brief Bioinform. 2017 Jan 3;19(2):350–8. doi: 10.1093/bib/bbw120 (PMC6018860; doi:10.1093/bib/bbw120)
Supplement: Supplementary Data [file bbw120_supplementary_file.pdf]

Supplementary Material

Design of RNAs: Comparing Programs for inverse RNA folding

Alexander Churkin, Matan Drory Retwitzer, Vladimir Reinharz, Yann Ponty, Jérôme Waldispühl, Danny Barash

1 Command Line Interfaces

1.1 `RNAinverse`

The command line interface of `RNAinverse` allows subtle optimizations while the main parameters are inserted upon software request. More advanced options exists for custom alphabet, energy parameters and base pairing. Those would not be discussed here as they are a very rare usecase. For the average user, the following are the ones that will be most used.

`-T` Rescale energy parameters for a given temperature.

`-F` Select the minimization algorithm. `m` for energy minimization or `p` for partition function.

`-R` The number of output sequence to output for the same structure. Negative number will force the software to continue until a perfect match.

`-noGU` Do not allow GU pairs.

`-noClosingGU` Do not allow GU pairs at the end of helices.

Therefore, to find a maximum of 50 solutions, using both partition and energy minimization algorithms, for the structure

```
((((...(((....)))...((((....))))...))))
```

allowing any sequence with a mandatory `GC` base between the first and last nucleotide, at 25 Celsius, the command would be as follows:

```
./RNAinverse -R50 -Fmp -T25
```

Once the software begins, it will request an input structure and starting sequence. Lowercase letters will be forced into the sequence while uppercase will be considered a starting sequence. If no sequence is inserted a random seed sequence will be used.

```
((((...(((....)))...((((....))))...))))
gNNNNNNNNNNNNNNNNNNNNNNNNNNNNNNNNNNNNNc
```

1.2 `incaRNAfbinv`

`incaRNAfbinv` is a combination of two seperate programs, both have specific command line interfaces. It is recommended for most users to use the web-server as it already combines the two interfaces.

`incaRNAtion` generates the seed sequences later inserted into RNAfbinv. To run it, a Python distribution must be installed. The command line interface includes many fine tuning parameters while the main structure input must be inserted in a file. The input file must contain a target structure. In addition to the structure, a multiple sequence alignment (MSA) may be added to allow for sequence information. For the average user, the following are the ones that will be most used.

`-d` The path for an input file containing secondary structure and optional MSA.

`-a` A number between 0 and 1 used by the algorithm as a weight. 1 takes into account on the structure while 0 only considers the MSA.

`-m` Maximum penalty for an invalid pair.

`-s_gc` This is followed by 2 numbers. The first, between 0 and 1, forces a given GC content while se second show the minimal number of output sequences required.

`-gc_max_err` A number between 0 and 1 with the maximal GC difference between the output sequences and the requested number. 0.1 by default.

-c Sequence constraint to output sequences

To generate at least 50 seed sequences, for the structure

```
((((...(((....)))...((((....))))...))))
```

allowing any sequence with a mandatory `GC` base between the first and last nucleotide and a `GC` content of 70%, should be done as follows.

First create a file `INPUT_FILE`, where `INPUT_FILE` can be admissible file name later given to the `-d` option, containing the line:

```
((((...(((....)))...((((....))))...))))
```

Then, call the `incaRNAtion` script:

```
python IncaRNAtion -d INPUT_FILE -a 1 \
   -m 20 -s_gc 0.7 59 \
   -c GNNNNNNNNNNNNNNNNNNNNNNNNNNNNNNNNNNNNNC
```

The output will be a list a seed sequences. Those seed sequences can later be insrted into `RNAfbinv` using the command line.

To use the `incaRNAtion` seeds, download the `RNAfbinv` extended version. The package includes a java GUI interface. The command line option allow for the same options as the GUI version. For the average user, the following are the ones that will be most used.

-i The number of simulated annealing iterations for a single sequence design.

-t Look ahead depth: The maximum number of consecutive mutations that generate a lower score sequence possible before a single simulated annealing iteration is over.

-c A starting sequence. This is where the `incaRNAtion` seed should be inserted.

To generate a single sequence, for the structure

```
((((...(((....)))...((((....))))...))))
```

allowing any sequence , starting from a given `incaRNAtion` seed, aiming at -23 dG (Kcal/mol) and target mutational robustness 0.8 given 50 iteration and a 4 nucleotide look ahead depth, the command should be a followed:

```
./RNAexinv -i 50 -t 4 -c <incaRNAtion seed>
```

Once the programs starts the following parameters should be inserted:

```
((((...(((....)))...((((....))))...)))) \
-23 \
0.8
```

1.3 RNAiFold

The command line interface of RNAiFold has over 50 options allowing for an extremely fine tuning of the desired output. For the average user, the following are the ones that will be most used. There is two way to enable those options, or through a file, where the option name is on a line preceded by a # instead of a -, followed on the next line by the desired option. Usually, the option can be simply given as argument on the command line.

-RNAscdstr The target structure. Multiple target can be set, they must be on the same line separated by the pipe | symbol. The structures must have the same length.

-RNAseqcon The admissible sequences, in IUPAC format. It must be one string the same length as the structure.

-maxGCcont The maximal GC content admissible in the sequences.

-minGCcont The minimal GC content admissible in the sequences.

-TimeLimit The amount of time allowed to run (default 600 seconds).

-MAXsol The maximum number of solutions to be reached under the time limit

Therefore, to find a maximum of 50 solutions, under an hour, for the structure

((((...(((....)))...((((....))))...))))

allowing any sequence with a mandatory GC base between the first and last nucleotide, and with a GC content between 60% and 70%, the command would be as follows:

```
./RNAiFold -RNAscdstr "((((...(((....)))...((((....))))...))))" \
           -RNAseqcon GNNNNNNNNNNNNNNNNNNNNNNNNNNNNNNNNNNNNNNC \
           -maxGCcont 70 -minGCcont 60 -MAXsol 50 -TimeLimit 3600
```

1.4 antaRNA

The distribution of antaRNA as a Python2.7 executable and all options are given as arguments. A similar ensemble of constraints exists.

`-Cstr` The target structure in the dot bracket notation. A fuzzy notation can be used to define blocks allowed to base pair together using any lowercase and uppercase letter.

`-Cseq` The admissible sequences, in IUPAC format. It must be one string the same length as the structure.

`-tGC` Target `GC` content, in $[0, 1]$, which also serves as a minimum.

`-tGCmax` Maximal `GC` content admissible in the sequences.

`-tGCvar` Variance (σ^2) in the case of normal distribution, `-tGC` serves as the expected value μ.

`-t` The amount of time allowed to run (default 600 seconds).

`-n` Number of solutions to be produced.

Therefore, to find a maximum of 50 solutions, under an hour, for the structure

```
((((...(((....)))...((((....))))...))))
```

allowing any sequence with a mandatory `GC` base between the first and last nucleotide, and with a `GC` content between 60% and 70%, the command would be as follows:

```
python antaRNA.py -Cstr "((((...(((....)))...((((....))))...))))" \
            -Cseq GNNNNNNNNNNNNNNNNNNNNNNNNNNNNNNNNNNNNNNC \
            -tGC 0.6 -tGCmax 0.7 -n 50 -t 3600
```

Pseudoknotted structures can be considered with the `-p` parameter if `pKiss_mfe` or `HotKnots` or `IPKnot` is installed,.

In addition, all parameters of the ant colony search algorithms can be directly modified through the command line, from the random seed to initiate the search `-s`, the number of ants exploring (`-aps`, default 10), the pheromone evaporation rate(`-er`, default 0.2), and a wealth of others.

1.5 NUPACK

The NUPACK program provides an ensemble of tools, design being the application for inverse folding. It has less options than the previous programs but with his focus for designing long sequences viable *in vitro*, it can extrapolate the energy parameters for a given concentration of sodium and magnesium.

The program loads the target structure and admissible sequences, in IUPAC format, from a file PREFIX.fold. The PREFIX can be any name chosen by the user but the extension .fold must be given. Additional parameters are:

-material which can be set as rna1995 to use Turner95 energy or rna1999 for Mathews99 energy parameters.

-sodium The sodium concentration.

-magnesium The magnesium concentration.

-prevent The name of a file, which can contain one subsequence per line forbidden in the design.

-loadseed PREFIX.init A file containing one number, the random seed to be used. Each execution of the software will choose a different random seed, but the program is deterministic and will always return the same output for a given seed. Note that the name of the file *must* be the same as the one with the target sequence, followed by the extension .init.

To design any sequence with a mandatory GC base pair between the first and last nucleotide, for the structure

((((...(((....)))...((((....))))...))))

using Turner95 energy parameters should be done as follows.

First create a file PREFIX.fold, where PREFIX can be admissible file name, containing the two lines:

```
((((...(((....)))...((((....))))...))))
GNNNNNNNNNNNNNNNNNNNNNNNNNNNNNNNNNNNNNC
```

Then, call the function design.

```
./design -material rna1995 PREFIX
```

Note that the suffix .fold is not given. To generate a different sequence launch the program again.
